# Comorbidities in hereditary angioedema—A population‐based cohort study

**DOI:** 10.1002/clt2.12135

**Published:** 2022-03-26

**Authors:** Linda Sundler Björkman, Barbro Persson, David Aronsson, Lillemor Skattum, Patrik Nordenfelt, Arne Egesten

**Affiliations:** ^1^ Respiratory Medicine & Allergology Department of Clinical Sciences Lund Lund University and Skåne University Hospital Lund Sweden; ^2^ Department of Immunology, Genetics and Pathology The Rudbeck Laboratory Uppsala University Uppsala Sweden; ^3^ Department of Laboratory Medicine Section of Microbiology, Immunology and Glycobiology Lund University and Clinical Immunology and Transfusion Medicine Lund Sweden; ^4^ Respiratory Medicine & Allergology Department of Internal Medicine County Hospital of Ryhov Jönköping Sweden

**Keywords:** autoimmunity, cardiovascular disease, complement, epidemiology, hereditary angioedema (HAE)

## Abstract

**Background:**

In hereditary angioedema (HAE), low levels (type 1) or defect in function (type 2) of the serine‐protease inhibitor C1 Inhibitor protein results in activation of the classical pathway of the complement system as well as the contact system. Here, we investigated the risk of comorbidities in HAE.

**Methods:**

Individuals with HAE (*n* = 239; identified through a physician made diagnosis) and a control cohort from the general population (*n* = 2383; matched for age, gender, and county of residence) were compared with the Swedish inpatient, cause of death, cancer, and prescription registers. Conditional logistic regression was used to analyze the data.

**Results:**

Increased risk of cardiovascular disease (odds ratio [OR] 1.83; 95% confidence interval [CI] 1.32–2.54), including arterial (OR 6.74; 95% CI 1.89–24.06) and venous thromboembolic disease (OR 4.20; 95% CI 2.42–7.23) as well as hypertension (OR 1.64; 95% CI 1.12–2.39) was seen in HAE. There was also an increased number of individuals diagnosed with hyperlipidemia (OR 2.01; 95% CI 1.16–3.50) among HAE patients. Furthermore, the risk of autoimmune disease was increased (OR 1.65; 95% CI 1.15–2.35) being particularly pronounced for systemic lupus erythematosus (OR 71.87; 95% CI 8.80–586.7). The risk of having two or more autoimmune diseases was also higher among HAE patients (*p* = 0.017). In contrast, the risk of cancer was not increased. Data from the prescription register revealed higher prescription rates of drugs against hypertension, hypothyroidism, and hyperlipidemia among HAE patients.

**Conclusions:**

The results warrant for awareness and prevention of comorbid conditions, in particular, thromboembolic and autoimmune diseases in HAE. Future prophylactic interventions may modify these risks.

## 1 INTRODUCTION

Individuals with hereditary angioedema (HAE) have an ongoing activation of the complement system due to mutations in the *SERPING1* gene. This gene encodes the serine‐protease inhibitor C1 inhibitor protein (C1‐INH) and the mutations result in either low levels of C1‐INH (HAE type 1) or a dysfunctional protein (HAE type 2). Patients experience episodes of angioedema, affecting cutaneous and submucosal tissues of various parts of the body.^1^, ^2^, ^3^ A feared consequence is edema of the upper airways where it may cause asphyxia.^4^

In addition to regulating the classical and lectin pathways of the complement system, C1‐INH is also the main regulatory protein of the contact system, inhibiting activated factor XII (FXIIa), kallikrein, and activated factor XI (FXIa). During angioedema attacks, there is an increased generation of thrombin, with signs of activation of both the contact and tissue factor coagulation pathways.^5^ Thus, chronic activation of the classical pathway of the complement system as well as the contact and coagulation systems may pave the way for comorbid conditions in HAE. Previous studies have shown a link between HAE and autoimmune diseases, in particular systemic lupus erythematosus (SLE).^6^, ^7^, ^8^, ^9^ Also, increased prevalence of other autoimmune disorders, including autoimmune thyroiditis, has been reported in HAE.^10^, ^11^ To the best of our knowledge, the prevalence of other dysregulated immune responses, for example, the prevalence of allergic disease and asthma, has not been investigated in HAE.

Treatments used to prevent angioedema in HAE may change the risk of various comorbid conditions. Currently used prophylactic treatment options in HAE include substitution with recombinant or plasma‐derived C1‐INH as well as treatment with attenuated androgens, mainly danazol or oxandrolone.^3^ Side effects of attenuated androgens can contribute to comorbidities in HAE since long‐term use of danazol is associated with increases in atherogenic indices.^12^, ^13^ Some studies also suggest that increased risks of myocardial infarction, stroke, deep vein thrombosis, and other cardiovascular abnormalities using treatment with androgens.^12^, ^13^, ^14^ The literature includes case reports of patients with liver cell adenoma and carcinoma associated with attenuated androgen use.^15^ Therefore, their use for prophylaxis in HAE has been questioned.^16^

This study set out to study comorbidities in HAE, using register‐based strategies.

## 2 MATERIALS AND METHODS

### 2.1 Setting

The health care in Sweden is publicly financed with few private care‐givers in secondary care. A personal identity number provides the possibility of cross‐referencing information in national databases. Prescription of pharmaceutical drugs from both primary and secondary care is registered in the National Prescription Register while there is no nationwide register for diagnoses in primary care.

### 2.2 Identification of a nationwide cohort of HAE patients and controls

By contacting most of the HAE‐treating physicians and by using databases at clinical immunology laboratories in Sweden, individuals diagnosed with either HAE type 1 or 2 were identified. The diagnoses were based on a history of angioedema and low levels of complement C4 in combination with either low levels of C1‐INH (type 1) or normal or increased levels but defect in function (type 2). By using the National Population Register at Statistics Sweden (SCB), a control cohort consisting of 2383 individuals (about 10 controls per HAE‐patient) from the background population was identified, matched for age, gender, and county of residence. Both HAE patients and controls were included in the study at the time of their date of birth. The two cohorts were compared with The Inpatient Register (*Patientregistret*), the Swedish Cause of Death Register (*Dödsorsaksregistret*), the Swedish Cancer Register (*Cancerregistret*), and the Swedish National Prescription Register (*Läkemedelsregistret*) that started in July 2005.

The inpatient register includes all diagnoses and hospital care as well as diagnoses from the outpatient care (using the International Classification of Diseases [ICD] [WHO], ICD‐9 [1987–1996], and ICD‐10 [1997–present]); the disease codes used are listed in supporting information S1). Validation studies of the inpatient register indicate that coverage is above 98% and that almost 85%–95% of the diagnoses reported are correct.^17^

### 2.3 Statistics

Odds ratios (OR) with 95% confidence intervals (95% CI) were calculated comparing the cases and controls by disease groups, and specific diseases. Conditional maximum likelihood estimation (Fisher) was used for *p*‐values when the number of events were small (*n* < 5). The Kaplan–Meier approach was used together with the log‐rank test to study time to first event for the specific outcomes of interest (autoimmune disease, allergy and asthma, cardiovascular disease (CVD), and cancer) by cases and controls. The corresponding Hazard ratios between the groups were calculated by using Cox regression models, and the assumption of non‐proportional hazards were met. Statistical significance was considered at 5% alpha level, and data management was done in SAS version 9.4 (SAS Institute, Cary, NC) and the statistical analysis was performed using R version 3.4.1 (R Foundation for Statistical Computing, Vienna, Austria). Linear mixed regression models were used in order to account for the dependency over calendar year when studying associations between cases and controls, and drug use expressed as proportions over calendar year. An interaction term was fitted to address potential changes in the outcome of interest.

### 2.4 Ethical considerations

The study was approved by the Swedish Ethical Review Authority (2019‐01,623) and executed in accordance with the Helsinki declaration.

## 3 RESULTS

### 3.1 Demographic characteristics and mortality

The study included 239 individuals with HAE, and for each HAE‐patient 10 individuals from the general population matched for age, gender, and county of residence (*n* = 2383) were identified in the Swedish Population Register (Table 1). In one case, it was only possible to identify three controls due to insufficient numbers of matching individuals in the county. Out of the 239 individuals, the majority had HAE type 1 (90.4%) while 9.6% where characterized as HAE type 2. Of the HAE patients included in the current study, 146 were previously part of the Swedish population‐based HAE registry (Sweha‐Reg), where individuals were included between 2007 and 2011.^18^, ^19^ The additional identification of HAE cases in this study increases the minimal HAE prevalence from 1.54 to 2.19 HAE cases/100 000 inhabitants in Sweden. The 239 individuals most likely represent the majority of all individuals diagnosed with HAE in Sweden since 2007. This, since the diagnosis is based on both a history of angioedema and repeated laboratory analyses of complement, information that was obtained from both physicians treating HAE in parallel with all laboratories performing such laboratory diagnostics in Sweden.

**TABLE 1:** Demographic characteristics of the study population

	HAE patients	Reference population^a^
	*n* = 239 (100%)	*n* = 2383 (100%)^b^
Male/female (100%)	111/128 (46%/54%)	1103/1280 (46%/54%)
Age (years)^c^		
0–19	49 (20.5%)	490 (20.6%)
20–39	67 (28.0%)	663 (27.8%)
40–59	66 (27.6%)	660 (27.7%)
≥60	57 (23.9%)	570 (23.9%)
*HAE type 1*	216 (90.4%)	
Male/female	102/114 (47%/53%)	
Age (mean; SD)	43; 23 years	
*HAE type 2*	23 (9.6%)	
Male/female	9/14 (39%/61%)	
Age (mean; SD)	39; 21 years	

Abbreviation: HAE, hereditary angioedema.

^a^ Control subjects were matched for year of birth, gender and county of residence.

^b^ For one HAE patient, only three control individuals could be identified.

^c^ At date of enrollment in the study (September, 2020).

### 3.2 Cardiovascular disease

**FIGURE 1:**
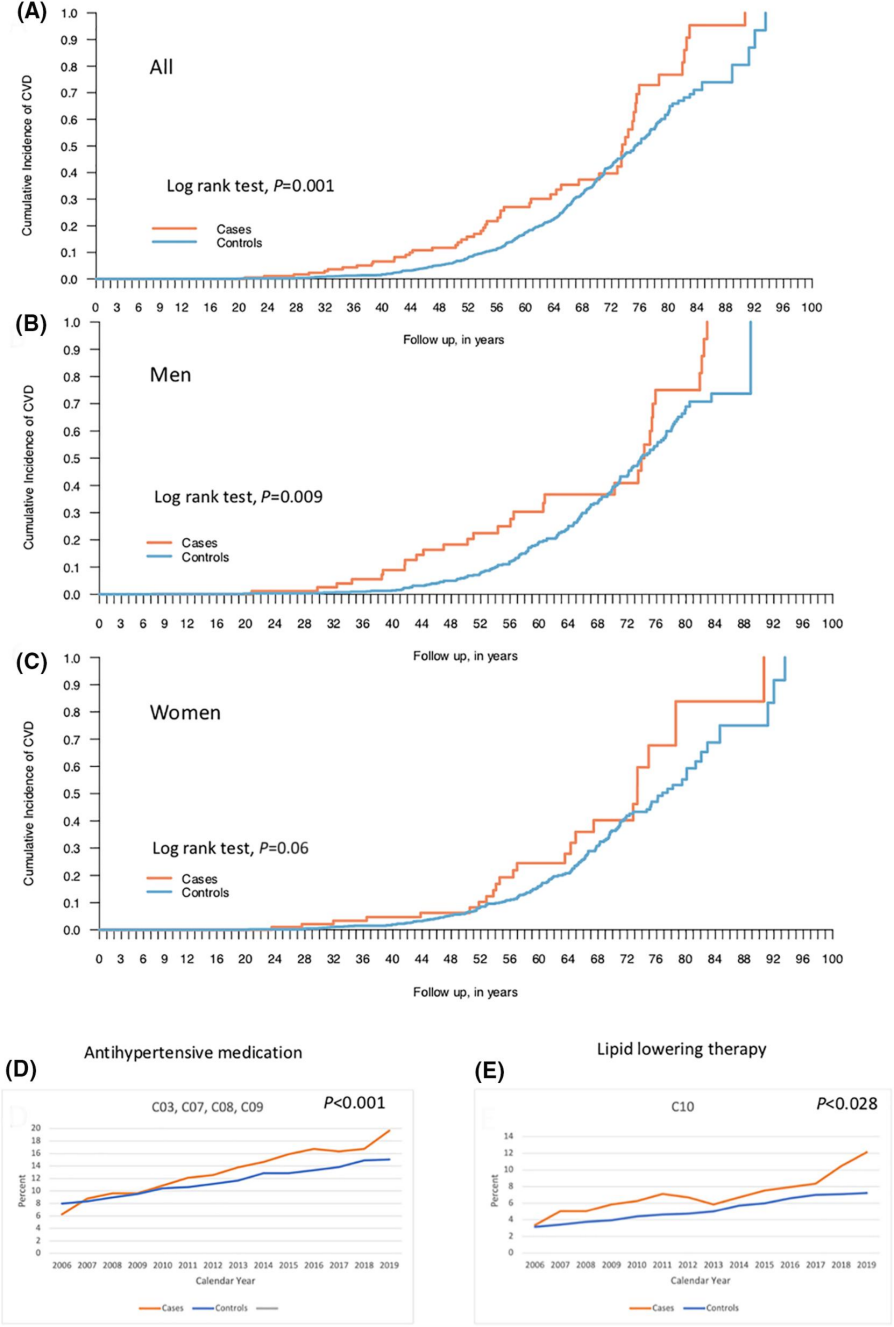
Cumulative incidence of cardiovascular disease comparing individuals suffering from hereditary angioedema (cases) and a background population (controls). All cases and controls (A), men (B), and women (C). Log rank test was used to determine statistical significance. Antihypertensive (D) and lipid‐lowering medication (E) prescribed among cases and controls, respectively, during the time period 2006–2019. Linear mixed regression models were used in order to account for the dependency over calendar year when studying associations between cases and controls and drug use expressed as proportions over calendar year. An interaction term was fitted to address potential changes in the outcome of interest

Cases and controls were compared with the Inpatient Register using the ICD‐9 and ICD‐10 classifications (Table 2). The risk of CVD was higher among HAE patients compared to controls (OR 1.83; 95% CI 1.32–2.54; Table 2). The cumulative incidence of CVD was significantly higher for the HAE cohort (*p* = 0.001) with differences being apparent between the third and sixth decades of life (Figure 1A). When subgrouping HAE patients based on gender, middle‐aged men (i.e. third to sixth decades of life) still had a higher risk (Figure 1B; *p* = 0.009) while there was no significantly increased risk for women compared with controls (Figure 1C; *p* = 0.06). In particular hypertension (OR 1.64; 95% CI 1.12–2.39) and thromboembolic events, both on the arterial (OR 6.74; 95% CI 1.89–24.06) and venous sides (OR 4.20; 95% CI 2.42–7.23) were significantly increased. In recent years, a significant higher portion (20%) of the HAE patients were prescribed antihypertensive medication (*p* < 0.001) compared with controls (15%; Figure 1D). Hyperlipidemia, a risk factor for CVD, was twice as common in HAE patients compared to controls (OR 2.01; 95% CI 1.16–3.50). A significantly higher prescription of lipid‐lowering therapy was also found for HAE patients (*p* = 0.028; Figure 1E).

**TABLE 2:** Cardiovascular diseases as comorbid conditions in HAE

Diagnosis	Cases	Controls	OR (95% CI)	*p* value
	*n* = 239 (100%)	2383 (100%)		
All cardiovascular diseases	53 (22.18%)	321 (13.47%)	1.83 (1.32–2.54)	<0.001
Arterial thrombosis/embolus	4 (1.67%)	6 (0.25%)	6.74 (1.89–24.06)	0.009
Cerebral infarction	4 (1.67%)	52 (2.18%)	0.76 (0.27–2.13)	0.604
Brain hemorrhage	1 (0.42%)	16 (0.67%)	0.62 (0.08–4.71)	0.642
Hypertension	36 (15.06%)	233 (9.78%)	1.64 (1.12–2.39)	0.013
Ischemic heart disease^a^	12 (5.02%)	98 (4.11%)	1.23 (0.67–2.28)	0.497
Pulmonary embolism	4 (1.67%)	21 (0.88%)	1.91 (0.65–5.62)	0.229
Venous thrombosis/embolus	19 (7.95%)	48 (2.01%)	4.20 (2.42–7.23)	<0.001
Deep vein thrombosis	4 (1.67%)	18 (0.76%)	2.24 (0.75–6.66)	0.138

Abbreviations: HAE, hereditary angioedema; ICD, International Classification of Diseases.

^a^ Including angina pectoris and acute myocardial infarction. The disease codes used in ICD‐9 and ICD‐10, respectively to identify cardiovascular co‐morbidities are listed in supporting information S1.

### 3.3 Autoimmune diseases

**FIGURE 2:**
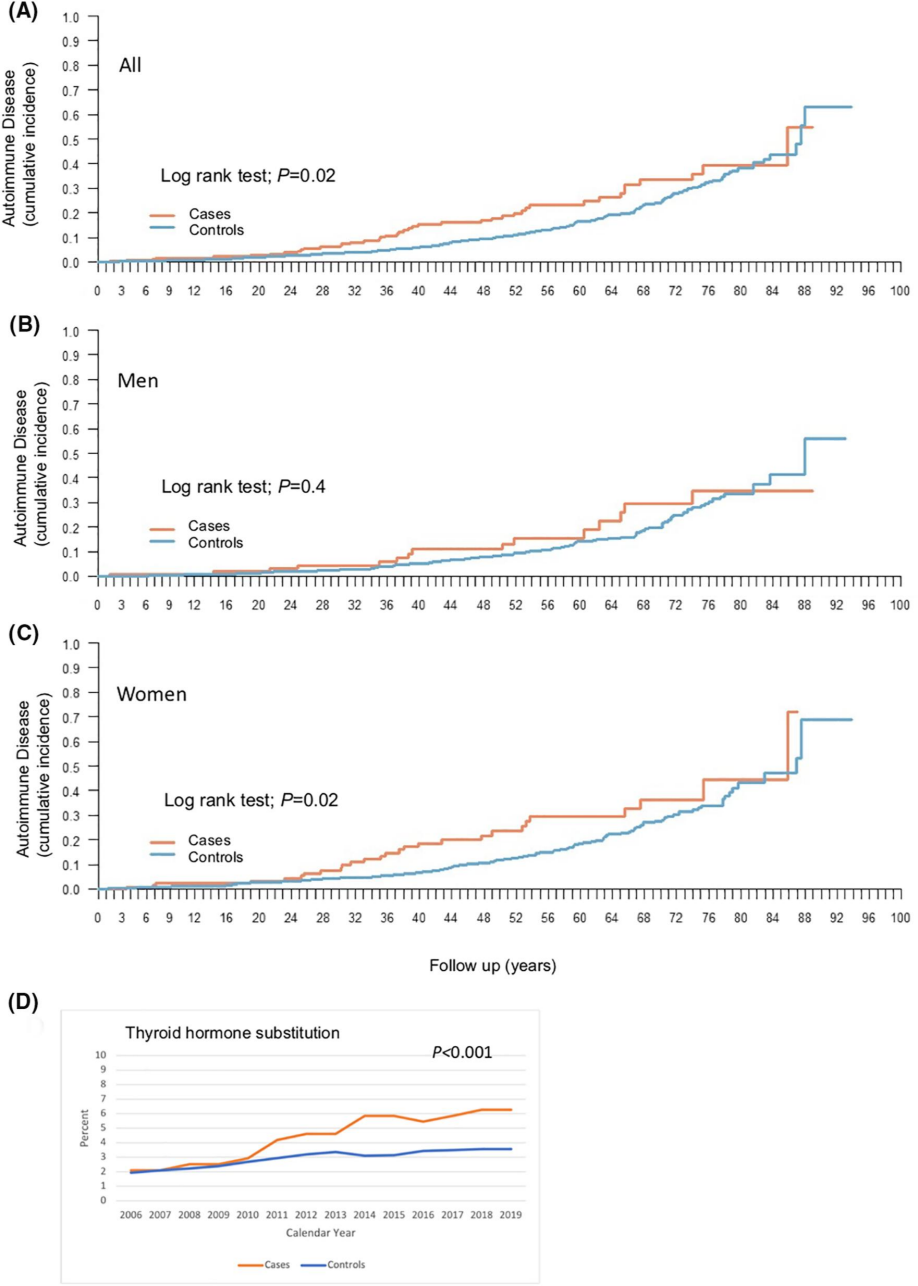
Cumulative incidence of autoimmune disease comparing individuals suffering from hereditary angioedema (cases) and a background population (controls). All cases and controls (A), men (B), and women (C). Log‐rank test was used to determine statistical significance. (D) Prescription of thyroid hormone substitution among cases and controls, respectively, during the time period 2006–2019. Linear mixed regression models were used in order to account for the dependency over calendar year when studying associations between cases and controls and drug use expressed as proportions over calendar year. An interaction term was fitted to address potential changes in the outcome of interest

Including all autoimmune diseases, HAE patients showed an increased risk (OR 1.65; 95% CI 1.15–2.35; Table 3). In subgroup analyses, this was also evident for endocrine autoimmune diseases (OR 1.98; 95% CI 1.18–3.35) as well as for SLE (OR 71.87; 95% CI 8.80–586.7). Using log rank test, the increased morbidity with regard to autoimmune diseases began in the second decade and lasted into the seventh decade of life (*p* = 0.02; Figure 2A). Here, men showed no significant cumulative incidence of disease (*p* = 0.4; Figure 2B) while it remained significant in the subgroup of women (*p* = 0.02; Figure 2C). In general, autoimmune hypothyroidism and SLE are more prevalent in women than in men.^20^, ^21^ This was also seen in the current study. In HAE patients with hypothyroidism, 22% were men and 78% were women. A similar distribution was found among controls with 17% being men and 83% being women. In HAE patients with SLE, 14% were men and 86% women while among controls, one woman and no men had a diagnosis of SLE. In addition, the prescription of thyroid hormone substitution was significantly higher among HAE patients compared with controls (*p* < 0.001; Figure 2D). The risk of having two or more autoimmune diseases was also higher among HAE patients than among controls (*p* = 0.017).

**TABLE 3:** Autoimmune disease in HAE

Diagnosis	Cases	Controls	OR (95% CI)	*p* Value
	*n* = 239 (100%)	2383 (100%)		
All autoimmune diseases	42 (17.6%)	273 (11.5%)	1.65 (1.15–2.35)	0.007
Blood and immune system^a^	1 (0.4%)	9 (0.4%)	1.11 (0.14–8.79)	0.922
Endocrine system^b^	18 (7.5%)	94 (3.9%)	1.98 (1.18–3.35)	0.02
Nervous system and the eye^c^	2 (0.8%)	23 (1%)	0.87 (0.20–3.70)	0.845
Gastrointestinal tract^d^	4 (1.7%)	56 (2.3%)	0.71 (0.25–1.97)	0.505
Skin^e^	9 (3.8%)	60 (2.5%)	1.52 (0.74–3.09)	0.251
Musculoskeletal system and connective tissue^f^	13 (5.4%)	56 (2.3%)	2.39 (1.29–4.44)	0.004
SLE	7 (2.9%)	1 (0.04%)	71.87 (8.80–586.7)	<0.001
Glomerulonephritis and nephrotic syndrome	2 (0.8%)	10 (0.4%)	2.00 (0.44–9.19)	0.362

Abbreviations: HAE, hereditary angioedema; SLE, systemic lupus erythematosus.

^a^ Idiopathic thrombocytopenic purpura, autoimmune hemolytic anemia, pernicious anemia, sarcoidosis, and IgG4‐related disease.

^b^ Diabetes mellitus type 1 (only from ICD‐10 since the diagnosis is not available in ICD‐9), hypothyrosis, thyrotoxicosis, autoimmune thyroiditis, Morbus Addison, and amyloidosis.

^c^ Multiple sclerosis, Guillain–Barré, myasthenia gravis, and iritis.

^d^ Chronic atrophic gastritis, celiac disease, Morbus Crohn, ulcerative colitis, autoimmune hepatitis, primary biliary cirrhosis, and primary sclerosing cholangitis.

^e^ Pemphigus, pemphigoid, psoriasis, alopecia areata, lupus erythematosus, and scleroderma.

^f^ Rheumatoid arthritis, psoriatic arthritis, juvenile arthritis, Churg–Strauss syndrome, granulomatous polyangiitis, giant cell arteritis, temporal artery arteritis, microscopic polyangiitis, SLE, polymyositis, systemic sclerosis, Sjögren's syndrome, mixed connective tissue disease, polymyalgia rheumatica, and morbus Bechterew. The disease codes used in ICD‐9 and ICD‐10, respectively, to identify autoimmune comorbidities are listed in supporting information S1.

### 3.4 Allergy and asthma

**FIGURE 3:**
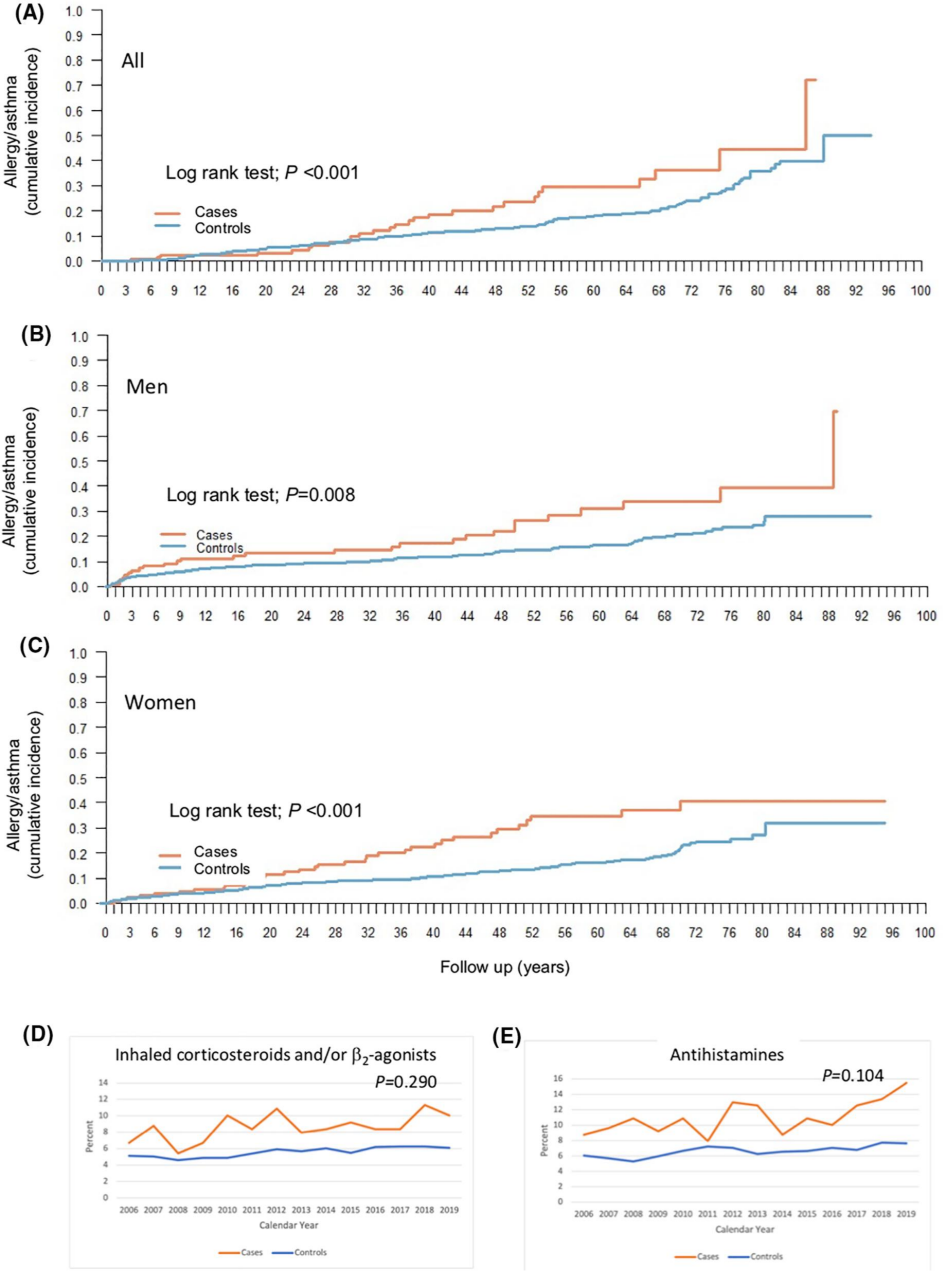
Allergic disease and asthma in hereditary angioedema. In total (A), men (B), and. women (C). Prescription of asthma medication (inhaled corticosteroids and/or b2‐agonists) (D) and antihistamines (E) prescribed among cases and controls respectively during the time period 2006–2019. Linear mixed regression models were used in order to account for the dependency over calendar year when studying associations between cases and controls and drug use expressed as proportions over calendar year. An interaction term was fitted to address potential changes in the outcome of interest

A diagnosis of allergy, asthma, or atopic dermatitis was based on codes in the Patient Register. No self‐reported diagnoses were included in this study. A two‐fold higher prevalence of allergy, asthma, and atopic dermatitis was found (OR 2.19; 95% CI 1.60–2.99). Furthermore, significantly higher cumulative incidences were seen for individuals suffering from HAE (*p* < 0.001), also when subgrouped into men (*p* = 0.008) and women (*p* < 0.001), respectively. However, a higher prevalence of asthma and allergic diseases could not be confirmed as reflected by a higher prescription of asthma medication (inhaled corticosteroids and/or β2‐agonists; *p* = 0.290) nor antihistamines for systemic administration (*p* = 0.104; Figure 3).

### 3.5 Cancer

In Sweden, a diagnosis of cancer is reported both by the treating clinician and the pathologist to the cancer register, rendering a high sensitivity and specificity. No increased risk of cancer was seen among HAE patients compared with controls (OR 0.89; 95% CI 0.57–1.42). Nor was the cumulative cancer incidence increased for HAE patients as a group (*p* = 0.4), or when subgrouped into men (*p* = 0.9) and women (*p* = 0.3) respectively (Figure S1).

### 3.6 Mortality

Historically, there was a high mortality, often at a younger age, related to HAE. In most cases this was caused by edema of the larynx resulting in asphyxia.^4^ 12 of the patients in the initial cohort (*n* = 146) were deceased at the time of the current data collection in 2019, an observation time of 8–12 years. One death was registered with HAE as the primary cause of death. In the current study, there was no increased risk of death (OR 0.69; 95% CI 0.38–1.26) among HAE patients. Using log rank test, the cohort of HAE patients displayed a longer time to death compared with controls (*p* = 0.048; Figure S2).

## 4 DISCUSSION

In this study, an increased risk of CVD, in particular arterial and venous thromboembolic disease as well as hypertension was found in patients with HAE type 1 and 2. It also confirms the results of previous studies, demonstrating an increased risk of autoimmune disease, in particular SLE and autoimmune endocrine disease. We did not see an increased risk of cancer.

The most important finding of the current study may be the increased risk of thromboembolic disease. An explanation may be that C1‐INH plays an important role regulating factor XII and factor XI, where insufficient levels of C1‐INH may trigger tissue factor induced activation of coagulation.^4^ Previous studies have shown a significant elevation in D‐dimer both in the symptom‐free period and a further increase during attacks in HAE‐patients, demonstrating activation of coagulation.^22^, ^23^, ^24^ Although elevated plasma D‐dimer levels are associated with acute HAE attacks, substitution with recombinant human C1‐INH therapy has not been associated with thrombotic events.^25^ However, neither increased risk of stroke (on the arterial side) nor pulmonary embolism was detected in this study. However, the diagnosis of pulmonary embolism is insidious; therefore, these figures may be falsely low. The risk for thromboembolic manifestations in HAE may also be balanced by activation of the fibrinolytic system. In parallel with coagulation, both fibrinolysis and potentiation of bradykinin‐production can be mediated by plasmin.^26^, ^27^, ^28^, ^29^ Furthermore, C1‐INH‐substitution prevents activation of both coagulation and complement, decreasing the D‐dimer levels.^29^

Both obesity and hyperlipidemia can occur as a result from long‐term treatment with attenuated androgens in HAE.^14^ Danazol can increase serum levels of low‐density lipoprotein cholesterol and reduce serum levels of high‐density lipoprotein (HDL) cholesterol.^30^ In a clinical trial, HAE patients treated with danazol for more than 2 years showed increased activation of coagulation, whereas shorter‐term treatment with danazol in healthy subjects in a clinical trial reduced HDL cholesterol levels but did not affect endothelial function or coagulation.^13^ In addition, low‐grade inflammation is important in the pathogenesis of CVD, not least atherosclerosis and recent concepts invoke inflammation as a key mediator between risk factors and artery wall cells.^31^ However, no increased risk of ischemic heart disease was observed here. In contrast, both higher prevalences of hypertension and hyperlipidemia as well as prescription of drugs against hypertension and hyperlipidemia was seen. This could be explained by a higher exposure to health care in the case of HAE patients, being subjected to increased attention to cardiovascular risk factors, thereby eliminating a higher risk by the disease itself or its treatments. The higher prevalence of CVD in men as compared to women in the HAE intra‐group analysis is intriguing. One possible explanation may be a higher vulnerability to low‐grade inflammation, adding to other risk factors of CVD in men. Risk factors for CVDs such as hypertension and hyperlipidemia are linked to body mass index BMI. However, the effects from BMI could not be adjusted for since the study was based on data from registers where information regarding BMI was not possible to obtain. Treatment of CVD, for example hypertension and heart failure, may also affect the course of HAE. There are no national guidelines in Sweden regarding treatment of hypertension in HAE patients. However, there is a general consensus not to use ACE‐inhibitors.

An increased prevalence of autoimmune diseases in HAE has been reported previously. In one study, 12% out of 157 individuals with HAE had autoimmune disease, including glomerulonephritis, Sjögren's syndrome, inflammatory bowel disease, thyroiditis, SLE, and rheumatoid arthritis.^7^ This is in the order of magnitude of the current study (17.6%). In particular, an increased risk of SLE was observed in the current study. In HAE, the lack of functional C1‐INH leads to chronic activation and consumption of C4.^3^ The resulting impaired classical pathway function in turn leads to a lack of C3b which is involved in the clearance of apoptotic cells which can result in production of autoantibodies. Autoantibodies and deposition of immune complexes into tissues are linked to several autoimmune diseases including SLE. Immune complexes have also been detected in HAE, possibly increasing the risk of this disease.^32^ Since many HAE patients are diagnosed and treated by allergists, a selection bias for a diagnosis (although likely to be accurate) of asthma and allergies in the HAE population as compared to controls is possible. On the other hand, several studies have reported over‐diagnosis of asthma in the general population, possibly diluting this effect.^33^ This may also be reflected by the higher prescription rates of drugs having these indications, although not reaching significant levels in the current study. In Sweden, the approved indications for prescribing antihistamines are allergic rhinitis and urticaria. It is unlikely that antihistamines are prescribed off‐label to treat HAE.

Long‐standing inflammation increases the risk of cancer as has been shown in several states of disease including sarcoidosis, rheumatoid arthritis, SLE, and ulcerative colitis.^34^, ^35^, ^36^, ^37^ However, the cancer risk was not elevated in the current study.

Disease activity in HAE, reflected as severity and frequency of angioedema episodes, may influence the risk of developing comorbidities. Since the study was based on codes from the patient register, such clinical information was not available. This important aspect should be analyzed in future, preferably, prospective studies. Another weakness of the study is that possible modifying effects from long term prophylaxis could not be investigated. In Sweden, attenuated androgens and tranexamic acid have been used off‐label for long term prophylaxis in HAE since the 1980s while plasma‐derived C1‐INH was approved for this use as of June 2011. However, the drug prescription register started out in July, 2005. As a result, the use of long‐term prophylaxis before this date is not possible to determine, making it difficult to draw conclusions regarding disease‐modifying effects from these in the cohort. Furthermore, discontinuation, use for varying time periods, and shifts between medications further makes it difficult to make valid conclusions. Also this needs to be investigated in future prospective studies, not least in the light of recent novel therapies becoming available.

The analysis of mortality in this current study is based on the 146 individuals suffering from HAE identified approximately 10 years ago and patients deceased before 2007 are not identified. In the current study another 93 individuals, identified through clinical immunology laboratories and treating physicians, were added. In contrast, living and deceased control individuals were followed from birth, most likely increasing the relative number of deaths in the reference population. Thus, the mortality rates of more than a decade is not possible to judge in the current HAE cohort. Nonetheless, the mortality among HAE patients during the last decade seems to be low. The increased attention by health care providers, providing health benefits, may be one explanation. Prospective studies should be important to follow up with regards to this aspect. Taken together, the results of this study points to the importance of awareness regarding thromboembolic and autoimmune disease among HAE patients. In particular among middle‐aged men with regard to CVD and among middle‐aged women with regard to autoimmune disease.

Future studies will be important regarding the effects on comorbidities from prophylactic treatments, for example, substitution therapy using C1‐INH‐concentrate or plasma kallikrein inhibitors.

## CONFLICT OF INTEREST

The authors declare no competing interests.

## AUTHOR CONTRIBUTIONS

**Linda Sundler Björkman:** Conceptualization; data curation; formal analysis; funding acquisition; software; supervision; writing – original draft; writing – review & editing. **Barbro Persson:** Data curation; resources; writing – original draft; writing – review & editing. **David Aronsson:** Conceptualization; Funding acquisition; Resources; Supervision; Writing – original draft; Writing – review & editing. **Lillemor Skattum:** Conceptualization; formal analysis; methodology; project administration; resources; supervision; writing – original draft; writing – review & editing. **Patrik Nordenfelt:** Conceptualization; formal analysis; resources; supervision; writing – original draft; equal; writing – review & editing. **Arne Egesten:** Conceptualization; funding acquisition; methodology; project administration; resources; supervision; writing – original draft; writing – review & editing.

## Supporting information

Supporting Information S1

FIGURE S1

FIGURE S2

## Data Availability

The data that support the findings of this study are available from the corresponding author upon reasonable request.
