# Supplementary material for: Comorbidities in hereditary angioedema—A population‐based cohort study
Source: Clin Transl Allergy. 2022 Mar 26;12(3):e12135. doi: 10.1002/clt2.12135 (PMC8967273; doi:10.1002/clt2.12135)
Supplement: Supplementary file 1 — Supporting Information S1 [file CLT2-12-e12135-s001.docx]

**Supplemental Material and Methods**

Disease codes from the International Classification of Diagnosis, ICD-9 and ICD-10 respectively, used to identify co-morbidities in the Swedish Inpatient Reister.

**Diagnosis** **ICD-9**  **ICD-10**

**Cardiovascular diseases**

Arterial thrombosis/embolus 444 I74

Cerebral infarction 433-434 I63

Brain hemorrhage 430-432 I61

Hypertension 401-405 I10-I15

Ischemic heart disease 410-414 I20-I25

Pulmonary embolism 415B I26-I28

Venous thrombosis/embolus 451, 452, 453 I80, I81,I82

DVT 451B I802

**Hyperlipidemia 272A-C E78**

**Allergy/asthma**

Allergy 477 J30

995A T782

995D T784

V150 Z910

Asthma 493 J45

Atopic dermatitis 691 L20

**Autoimmune diseases**

**Blood and immune system**

Idiopathic thrombocytopenic purpura 2873 D69.3

Autoimmune hemolytic anemia 283A D59.1

Pernicious anemia 281A D51.0

Sarcoidosis 135 D86

IgG4-related disease - D89.8A

**Endocrine system**

Diabetes mellitus type 1 - E10

Hypothyreosis 244 E03

Thyreotoxicosis 242 E05

Autoimmune thyreoiditis 245 E06.3

Morbus Addison 255E E27.1

Amyloidosis 277D E8

**Nervous system and eye**

Multiple sclerosis 340 G35

Guillain-Barré 357A G61.0

Myasthenia gravis 358A G70.0

Iritis 364A-D H20

**ICD-9 ICD-10**

**Gastrointestinal tract**

Chronic atrophic gastritis 535B K29.4

Celiac disease 579A K90.0

Morbus Crohn 555 K50

Ulcerative colitis 556 K51

Autoimmune hepatitis - K75.4

Primary biliary cirrhosis 571G K74.3

Primary sclerosing cholangitis 576B K83.0

**Skin**

Pemphigus 694E L10.0

L10.9

Pemphigoid 694F L12.0

L12.9

Psoriasis 696A-B L40

Alopecia areata - L63.9

Lupus erythematosus 695E L93

Scleroderma 701A L94.0

**Musculosceletal system and connective tissue**

Rheumatoid arthritis 714 (A, C, and W) M05-M06

Psoriatic arthritis **-** M07

Juvenile arthritis 714D M08

Churg-Strauss syndrome 446F M30.1

Granulomatous polyangiitis 446E M31.3

Giant cell arteritis - M31.5

Temporal artery arteritis - M31.6

Microscopic polyangiitis 446G M31.7

SLE 710A M32

Polymyositis 710E M33.2

Systemic sclerosis 710B M34

Sjögren´s syndrome 710C M35.0

Mixed connective tissue disease - M35.1

Polymyalgia rheumatica 725X M35.3

Morbus Bechterew 720 M45

**Glomerulonephritis and nephrotic syndrome**  580-582 N00-N05
