# Supplementary figures and images for: Comorbidities in hereditary angioedema—A population‐based cohort study
Source: Clin Transl Allergy. 2022 Mar 26;12(3):e12135. doi: 10.1002/clt2.12135 (PMC8967273; doi:10.1002/clt2.12135)

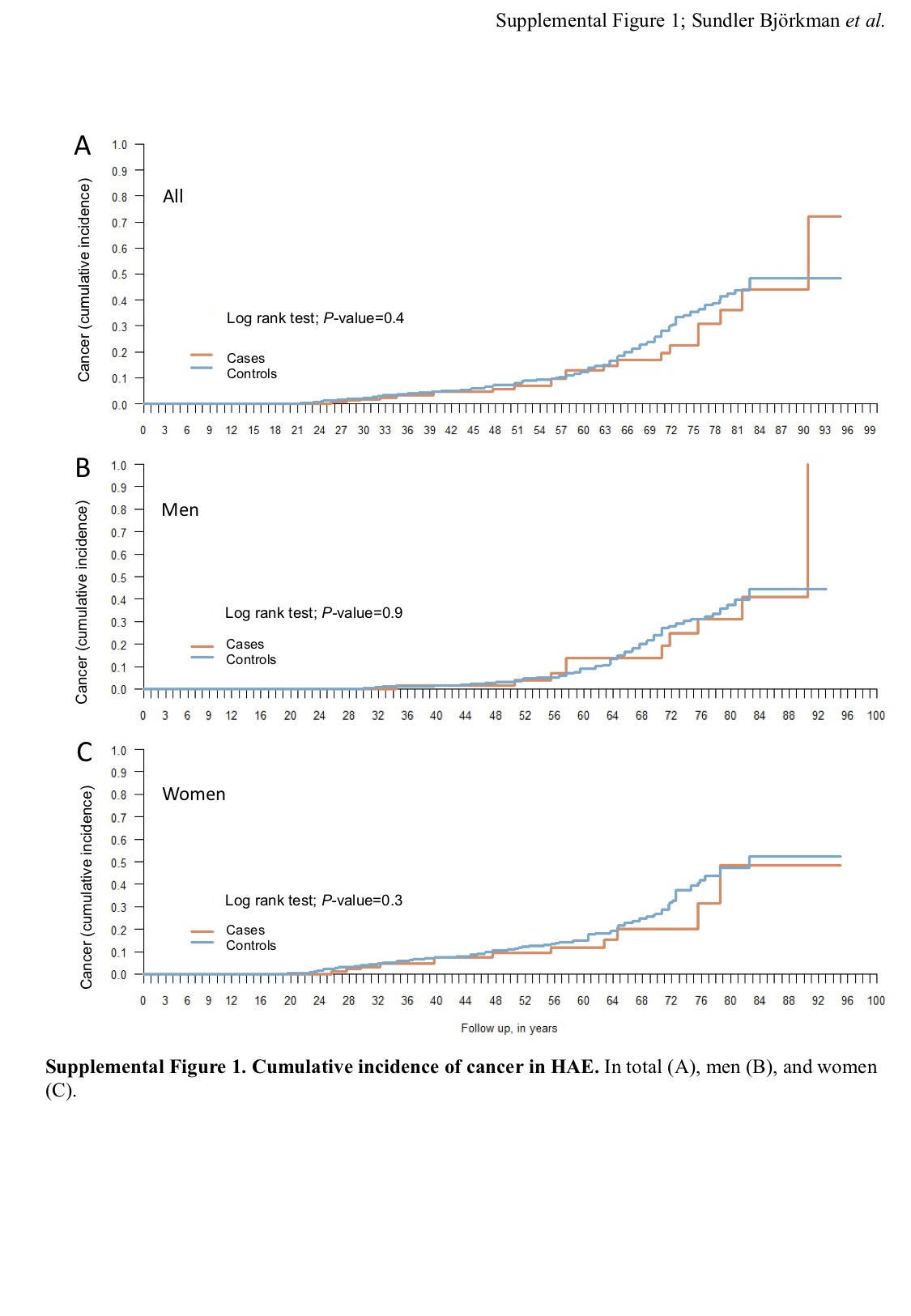

Supplement: Supplementary file 2 — FIGURE S1 [file CLT2-12-e12135-s003.png]

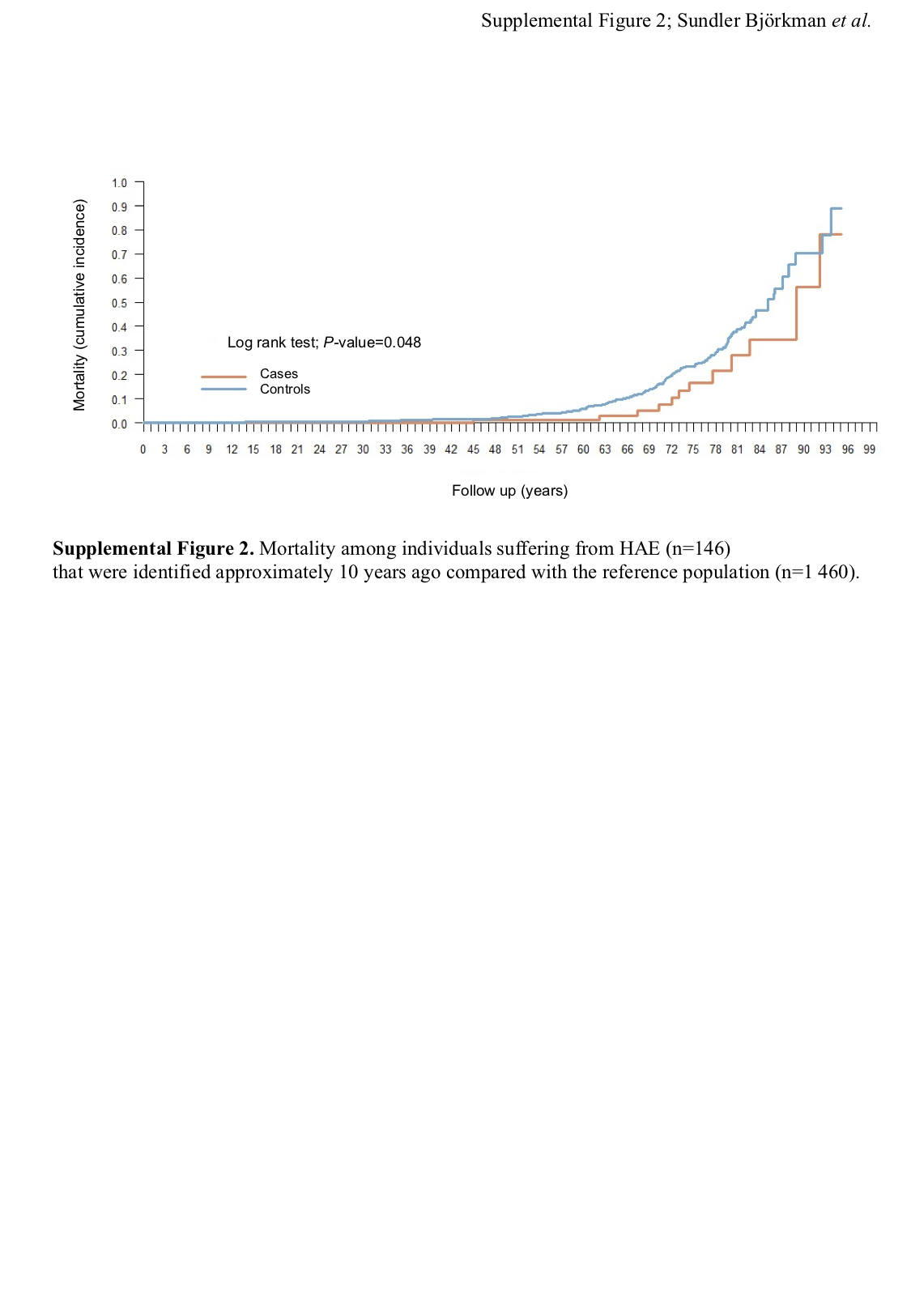

Supplement: Supplementary file 3 — FIGURE S2 [file CLT2-12-e12135-s002.png]
